# Cerebral Palsy in 1-12 Year Old Children in Southern Iran

**Published:** 2016

**Authors:** Soroor INALOO, Pegah KATIBEH, Masroor GHASEMOF

**Affiliations:** 1 Pediatric Neurologist, Neonatology Research Center, Shiraz University of Medical Science, Shiraz, Iran; 2 Pediatric Neurologist, Department of Pediatric, Shiraz University of Medical Science, Shiraz, Iran; 3 Pediatrician, Department of Pediatrics, Shiraz University of Medical Science, Shiraz, Iran

**Keywords:** Cerebral palsy, Hypoxic-ischemic encephalopathy, Risk factors, Iran

## Abstract

**Objective:**

Cerebral palsy (CP) is a non-progressive CNS disorder due to an insult to the growing brain, usually occurring in the first two years of life. During the recent years, its etiology has been changed; perinatal and postnatal insults are not considered as its main causes in developed countries any more. The aim of this study was to evaluate the causes of CP in children in southern Iran.

**Materials & Methods:**

Overall, 200 children with CP aged 1-12 yr old referring to Pediatric Neurology Clinic affiliated to Shiraz University of Medical Sciences, Shiraz, Iran between 2012 and 2013 were enrolled. In addition, 200 healthy age and sex-matched children were considered as the control group. Exclusion criteria were isolated movement disorders with no other evidence of CP, progressive neurologic disorders, metabolic disorders, and incomplete or uncertain past history. After collecting the data on pregnancy period, prenatal history and past medical problems, they were analyzed with appropriate statistical methods.

**Results:**

Maternal age, medical problems during pregnancy period, route of delivery, head circumference at birth, neonatal admission, neonatal jaundice, and prematurity were the main risk factors for CP.

**Discussion:**

The distribution of risk factors of CP is different from that of developed countries in our region. Pre- and peri-natal etiologies are still among the common causes of CP in Iran.

## Introduction

Cerebral palsy (CP) is a neurodevelopmental disorder usually occurring early in life and persisting thorough the life. According to the last proposed definition and classification of cerebral palsy “CP describes a group of permanent disorders of movement and posture causing activity limitation attributed to non-progressive disturbance in the developing fetal or infant brain”. Motor disorders are often accompanied by disturbance of sensation, perception, cognition, communication, and behavior as well as by epilepsy and secondary musculoskeletal problems (1). Risk factors of CP have been investigated, and can be categorized into three groups of prenatal, perinatal and postnatal ones. Prenatal factors can be summarized as follows: male gender, exposure to toxins such as methyl mercury during gestation, iodine deficiency, low or advanced maternal age (35 years), multiparity, nulliparity, previous dead fetus, multiple gestations, inappropriate inter-pregnancy interval, fever during pregnancy, preeclampsia and intrauterine growth retardation (2). Other related maternal factors such as irregular menstruation, epilepsy, diabetes, and thyroid disease are related to CP. It is more prevalent among those children whose siblings had suffered from CP during infancy or in the children born with assisted fertilization such as in vitro fertilization (3-4). Among perinatal factors, premature birth, abruptio placentae, low APGAR, low birth weight, intrauterine (TORCH) infections, and chorioamnionitis seem to have the most critical role (5). Post-natally, meningoencephalitis, kernicterus, intracranial bleeding, neonatal convulsion, sepsis, respiratory distress and genetic disorders can predispose the child to CP (6). Risk factors of CP have been listed differently in developing and developed countries. In Pakistan, the most prevalent cause of CP was birth asphyxia as 36% (7). Other etiologies were meningoencephalitis (34%), prematurity and low birth weight (8%), kernicterus (5.5%) and intracranial bleeding (2.5%); however, in 14% of the patients no specific reason was declared (idiopathic). In Turkey, asphyxia (39%), neonatal convulsion (34%) and kernicterus (19%) were the most prevalent causes of CP (8). A major risk factor of CP in Iran is asphyxia according to imaging studies recently available, such as MRI (9-12). On the other hand, in Australia, Sweden, United Kingdom and United States, congenital brain malformation, fetal intrauterine infections, genetic disorders and different toxin exposures account for the reason for CP in about 75% of cases (2, 3, 6). Peri-natal causes such as birth asphyxia, hypoglycemia, kernicterus and neonatal infections occurred in 6-8% of CP cases. However, postnatal risk factors such as seizure, sepsis and respiratory distress had a less prominent role (2, 3, 6). In America and Sweden, antenatal factors causing abnormal development of the brain consisted 80% of cases, and about 10% of the patients had evidence of intra-partum and birth asphyxia (3, 13). There are various problems in investigating the risk factors of CP such as delayed recognition, inappropriate considering of congenital malformation as hypoxic perinatal insults, inaccessible accurate data about birth history along with disagreements among examiners. However, this study was designed to have a more precise data in order to improve health care in Iran.

## Materials & Methods

Overall, 200 patients aged 1-12 yr old with previously confirmed diagnosis of CP, according to international definitions (1-2), who were under routine follow up in Pediatric Neurology Clinic (affiliated to Shiraz University of Medical Sciences, Shiraz, Iran) from 2012 to 2013 were enrolled into the study. The sample size was determined by a statistician according to previous similar studies. The case group was compared with the age and sex matched normal control group. The inclusion criteria of the case group were age between 1 to 12 yr with non-progressive disturbance of movement and posture along with defect in sensation, perception, cognition or communication. Isolated movement disorders with no other evidence of CP, progressive neurologic disorders, metabolic disorders, and incomplete or uncertain past history were considered as the exclusion criteria. The children in the control group, sex and age matched with the case group had completely normal neurologic and physical examination and were referred to the pediatric clinic for routine checkup or common childhood infection such as diarrhea or common cold. If there was any doubt about having abnormal neurologic examination in favor of CP, they were excluded from the study. All the patients in the case and control groups were examined by one pediatric neurologist and after convincing the parents for entering the study a questionnaire was filled out about the history of the patients. In the case group, the patient’s medical records were also reviewed and data about pregnancy and its complications, hospital admission, delivery or postnatal problem were extracted. After completing the questionnaires about detailed history of pre- and preinatal complications (History of asphyxia, bilirubin encephalopathy, RDS, congenital brain anomalies, meningitis, hypoglycemia, TORCH, multiple anomalies, admission due to unknown reason), special lab data or imaging (Brain MRI or CT scan) were ordered if indicated for diagnosis or management of the patients’ clinical manifestations. The study was approved by Ethics Committee of Shiraz University of Medical Sciences. Informed consent was taken from subjects’ parents. The data of this case-control study were analyzed using SPSS software version 15 (Chicago, IL, USA). Chisquare test and t-test were used to compare the case and control groups’ data. P value less than 0.05 was considered as statistically significant.

**Table 1:** Complications during Pregnancy and Delivery in the Case Group

**Complications**	**Number**	**Percentage**
**RDS**	12	6
**PROM**	13	6.5
**Pre-eclampsia**	6	3
**Gestational DM**	4	2
**Maternal thyroid disorder**	4	2
**Poly or oligohydraminos**	4	2
**Repeated abortions**	1	1
**Total**	45	22.5

**Table 2:** Distribution of Different Risk Factors in the Case and Control Groups

	**Risk factors**		**Case group** n (%)	**Control Group** n (%)	***P*** **-value**
**Prenatal risk factors**	Order of birth	First child	135(67.5 )	134(67 )	0.746
		Second to sixth child	65(32.5 )	66(33 )	
	Complication during pregnancy	yes	44(22 )	1(0.5 )	0.000
		no	156(78 )	199(99.5)	
	Microcephaly at birth	yes	10(5 )	0	0.006
		no	190(95 )	200(100 )	
	Living place	Urban	166(83 )	135(67.5 )	0.000
		Village	33(16.5 )	63(31.5 )	
		Suburban	1(0.5 )	2(1 )	
	Maternal age	Under 20 yrs	4(2 )	7(3.5 )	0.397
		20-35 yrs	182(91 )	186(93 )	
		Over 35 yrs	14(7 )	7(3.5 )	
	Type of pregnancy	Planned	181(90.5 )	188(94 )	0.107
		Unwanted	15(7.5 )	12(6 )	
		Assisted	4(2 )	0	
	Birth weight	Normal weight	190(95 )	198(99 )	0.02
		IUGR	10 (5 )	2(1 )	
**Perinatatal factors**	Route of delivery	Normal vaginal	107(53.5 )	152(76 )	0.000
		Abnormal vaginal	2(1 )	0	
		C/S	91(45.5 )	48(24 )	
	Prematurity	Preterm labor	50(25 )	18(9 )	0.004
		Term labor	150(75 )	182(93 )	
**Postnatal factors**	Site of admission	Neonatal emergency	84(42 )	22(11 )	0.000
		No admission	92(46 )	166(83 )	
		Neonatal intensive care unit	24(12 )	12(6 )	
	Management for hyperbilirubinemia	Exchange transfusion	23(12 )	0	0.000
		Phototherapy	52(26 )	33(16.5 )	
		No treatment	125(63 )	167(83.5 )	

## Results

The mean age of the case group was 2.7±2.2 (range from 1 to 12 yr) and in the control group, it was 2.2±1.7 yr. The patients of case and control groups were equally divided into 51% male and 49% female groups (P value: 0.4). Spastic CP was the most prevalent type of CP in the case group (61%). The most common form was spastic quadriplegia (Table 1). Prenatal, perinatal and postnatal risk factors are demonstrated in Table 2. In 35% of the patients, no clear etiologic factors were revealed.

## Discussion

During recent years, pre- and peri-natal health care has been improved dramatically; as the result, asphyxia and other perinatal complications are no longer prevalent as previous years. However, premature and low birth weight neonates survive more. Therefore, the net incidence of CP, in fact, does not seem to be reduced but the etiologies have been changed (3, 14). Maternal age is one of the most important factors; age more than 35 yr and less than 20 yr is considered a risk factor for having a CP child (3). The mean maternal age in the CP group was significantly lower than that of the control group (8). In our study, there was no significant difference between three categories according to maternal age (less than 20, 20-35 years and more than 35 years), but in the case group the incidence of maternal age more than 35 yr was 2 times that of the control group; also, in Iran maternal age>35 years was significantly associated with CP (12). So it might be concluded that extreme of maternal age is associated with greater risk of CP. Sex might be considered as a risk factor for CP (2, 15). In Turkey, CP was twice more prevalent in the males (8), but in our study and in North Iran (12) there was no significant difference as to sex. Jacobson in Sweden (2) reported CP to be more prevalent in high parity and nulliparity, but in this study, and North Iran (12) and Turkey (8) no association was detected between the number of pregnancy and CP. Most patients of the case and control groups in our study were the first or second child of the family, perhaps because recently most families have one or two children. CP was not significantly related to the order of birth (P value=1. 00). Like some other studies (3, 8), IUGR was a risk factor of CP in our study. Living in villages is supposed to be a risk factor for CP (8), but in the present study, CP had significantly higher prevalence in cities compared to village (P value: 0.00); probably appropriate health care of neonates and mothers in villages due easier access to family physician and earlier referral can account for this finding (urban family physician program was not yet carried out in our country at the time of study). Microcephaly as a risk factor for CP has an incidence of 0.56 to 2.3% at birth (8, 14). In this study, 5% of the patients in the case group had microcephaly, which was significant in comparison to the control group with normal head circumference. Preterm delivery is one of the most important risk factors for CP; even in neonates born in the 37th or 38th weeks of gestation, CP is more prevalent than neonates with gestational age of 40 weeks are. The incidence of preterm delivery in our study was similar to previous study (16). Premature delivery due to various complications was considered as a significant risk factor (P value=0.002). However, in Pakistan and Turkey, prematurity was not considered as an important risk factor for CP; this might be explained by lesser survival of premature neonates in these two countries (7, 8). Cesarean section (C/S) in this study was revealed to be twice more prevalent in the case group and is supposed to increase the risk of CP compared to normal vaginal delivery; however, in developed countries and Turkey, route of delivery was not considered as a risk factor (3, 8). The reason of this difference may be the fact that in our country, prolonged complicated delivery and fetal distress are among the most common indications for C/S. Better monitoring of the delivery rooms and earlier referral for C/S when indicated can prevent fetal distress and CP. Family history of CP is one of the risk factors (3) but in our study, this was not concluded. Maternal complication during pregnancy such as premature rupture of the membrane, pre-clampsia, placental abruption and thyroid disorders are important risk factors of CP (3,7). In our study, premature rupture of the membrane, pre-eclampsia, thyroid disorders, maternal DM, repeated abortions and prolonged labor had a higher incidence in the case group which was statistically significant (P value=0.002). In the present study, spastic CP was the most prevalent type of CP (61%) and quadriplegic spastic CP had an incidence of 36%. Other types of CP in this study were hypotonic (28%), paraplegic (20%), hemiplegic (5%), ataxic (5%) and athetoid (4%), nearly similar to what Nazir reported in Pakistan (7). In developed countries, the most important causes of CP are prenatal risk factors in 70% of cases (3, 17); in our study, 10% of the cases had CP due to prenatal causes. In addition, in Turkey and Pakistan congenital CNS anomalies, TORCH, infection, and genetic syndromes were not among the prevalent etiologies of CP (7, 8). Asphyxia was reported in 22% of patients in the present study, 10% in developed countries (3), 40% in Turkey (8) and 36% in Pakistan (7). History of neonatal emergency room and neonatal intensive care unit admission increased the risk of CP significantly compared to no admission (Table 2). Bilirubin encephalopathy was detected in 11.5% of the patients in this study, 20% in Turkey (8) and 5.5% in Pakistan (7), whereas no case was reported as a possible result of this problem in developed countries (3).The patients undergoing exchange transfusion had significantly higher incidence of CP compared to the patients treated with other methods of management for hyperbilirubinemia (Table 2). Early discharge from hospital after delivery, late referral for neonatal jaundice, poor compliance and reliance on traditional treatment in developing countries may account for this result. Meningitis (3%) was not as prevalent as Pakistan (34%) (7) in our study. In our study, 35% of the patients in the case group had no significant birth history and no hospital admission; however, they suffered from CP and were categorized as idiopathic group (the same as 14% of cases in Pakistan) (7). Four percent of CP patients in our study had a history of hospital admission in neonatal period without any definite diagnosis. This emphasizes for better investigation of probable risk of CP by considering more detailed laboratory and if indicated genetic studies for the patients categorized in the idiopathic group. However, other differential diagnosis of CP and slowly progressive disease mimicking CP should be re-evaluated. The most prevalent associated problem with CP was mental retardation (46%), reported by Liptak et al. as 50% (18). Seizure was reported in 16% of CP patients in our study but Kulak et al. reported it as 41% (19). Hearing defect was reported in about 10% of our CP patients, nearly similar to what Pharaoh et al. reported as 12% (20). Other problems of CP patients in the present study were constipation (10%), growth delay (3%), and visual disorders (3%). In conclusion, the most important risk factors of CP were route of delivery (C/S), maternal complications in pregnancy, neonatal Jaundice, microcephaly at birth, premature birth, neonatal hospital admission, and area of living, but sex, intrauterine growth retardation, maternal age and multiparity were not associated with increased risk of CP. Most of the causes of CP are preventable perinatal risk factors. Considering the fact that, in the south of Iran, the most common cause of seizure is perinatal brain insult (21), better health care during pregnancy, delivery and postnatal period is mandatory.
